# Prevalence of Eye Problems among Young Infants of Rohingya Refugee Camps: Findings from a Cross-Sectional Survey

**DOI:** 10.3390/tropicalmed5010021

**Published:** 2020-02-04

**Authors:** AHM Enayet Hussain, Zunayed Al Azdi, Khaleda Islam, ANM Ehtesham Kabir, Rumana Huque

**Affiliations:** 1 Ministry of Health and Family Welfare, Dhaka 1000, Bangladesh; paedeye@yahoo.com; 2 ARK Foundation, Dhaka 1212, Bangladesh; 3 Public Health Specialist, Dhaka 1206, Bangladesh; dr.khaleda.islam@gmail.com; 4 Directorate General of Health Services, Dhaka 1212, Bangladesh; dr.ehtesham.kabir@gmail.com

**Keywords:** pediatric eye problem, eye care, infant, Rohingya refugee

## Abstract

Early detection of pediatric eye problems can prevent future vision loss. This study was to estimate the prevalence of common eye problems among infants born in a resource-constrained emergency setting with a broader aim to prevent future vision loss or blindness among them through early detection and referral. We conducted a cross-sectional survey among 670 infants (0–59 days old) born in Rohingya refugee camps in Bangladesh between March and June of 2019. The most common eye problem found was watering from the eye and accumulation of discharge by which 14.8% of the children were suffering (95% CI: 12.2–17.7). More than 5% of the infants had visual inattention (95% CI: 3.5–7.0), and 4% had redness in their eyes (95% CI: 2.7–5.8). Only 1.9% of infants (95% CI: 1–3.3) had whitish or brown eyeballs, and 1.8% of children might have whitish pupillary reflex (95% CI: 0.9–3.1). None of the eye problems was associated with the gender of the infants. The prevalent eye problems demand eye care set up for the screening of eye problems in the camps with proper referral and availability of referral centres with higher service in the districts.

## 1. Introduction

A global initiative launched in 1999 named VISION 2020: The Right to Sight recognised blindness in children as a priority area of disease control [1]. In 2001, it was estimated that in low- and middle-income countries, more than half a million children with severe visual impairment and blindness had avoidable causes [2]. Later in 2010, a global estimate showed that around approximately 17.5 million children were at risk of developing low vision [3]. Childhood blindness can be caused by some common pediatric eye problems such as cataract, amblyopia, childhood tearing, and ptosis during infancy. The importance of prevention of childhood blindness is related to Disability Adjusted Life Years (DALY). Early detection of these is considered to be effective to avoid visual handicap worldwide.

In a recent survey, the prevalence of childhood blindness in rural Bangladesh was observed to be as 6.3 per 10,000 children [4], according to a national case series study, more than two-thirds of such cases could be avoided [5]. A qualitative study in Bangladesh identified barriers that influence eye care provision for children, which showed that barriers to early detection of symptoms, eye examination, and referral services could lead to permanent blindness in children which can be prevented by first empowering communities to recognise childhood cataract and take action [6]. A national campaign launched in 2004 found more than thirty-two thousand children as blind in Bangladesh, and highest number of those cases were found through key informant (KI) method which worked by providing short training to local volunteers to detect cases and refer to the health centres [7]. Also, a validation study found that in low-income settings, KI was an effective and a low-cost method for identification of cases with disabilities and visual impairment, KI was highly sensitive (100%) and specific (69%) [8]. Therefore, similar approaches can be applied to identify cases of common pediatric eye problems in order to ensure their early detection and treatment.

The largest refugee camp in the world is now located in Bangladesh, with around 915,000 Rohingya population [9]. These enormous numbers of displaced people have limited access to healthcare with higher health risks [10]. Evidence suggests that the prevalence of vision impairments and blindness among refugees are common and often higher than the general population. A recent systematic review found that the prevalence of blindness in the refugee camps can range from 1.3% to 26.2% [11]. A study with Afghan refugees in Pakistan revealed that 2.1% of all refugees there were blind, and 6.9% were visually impaired [12]. Also, a study in Uganda concluded that in refugee settlement camp setting, the prevalence was much higher than outside [13]. These pieces of evidence demand urgent attention to look into this issue in the refugee camps in Bangladesh. Studies show that the major interventions, in most places, including refugee camps, to control childhood blindness are public health in nature (vitamin A supplementation and measles immunisation) [14]. However, the need for eye care interventions for refugees are unique, and in every stage of displacement, such interventions should be targeted [11].

Childhood blindness causes a significant economic burden on the family and community [15]. In an extremely resource-constrained setting as refugee camps, this disease burden poses additional pressure to the government of the country and donors working to improve the health of such displaced people. In Bangladesh, it is a common problem that, parents often do not recognise the eye problems of their children and seek eye care in time. This study aimed to identify common eye problems among young infants born in refugee camps by trained refugee volunteers. The broader aim was to set an evidence-based ground that would help assess the need for eye care facilities in the camps. We conducted a cross-sectional survey among Rohingya infants during March– June of 2019.

## 2. Materials and Methods

### 2.1. Population

The target population for this study was 0–59 days old infants born and raised in the refugee camps. In Bangladesh, the integrated management of childhood illness (IMCI) protocol implemented at Primary Health Care (PHC) setting has incorporated eye component for early detection and prevention of childhood blindness. The IMCI protocol is divided into two components: one for age group 0 to 59 days, and another for 2 months to 5 years; for each set, there are age-specific eye components. For early screening and eye care provision, we chose the 0 to 59 days age group. Also, the Non-Communicable Disease Control Program (NCDC) of Directorate General of Health Services (DGHS), Ministry of Health and Family Welfare (MOHFW), with the technical assistance of World Health Organization (WHO) developed a training manual for health care providers working at PHC setting [16].

Based on a study done in 2018, the birth rate inside the Rohingya refugee camp is 35.6 per 1000 population [17]. The population of Rohingyas, according to UNHCR data [10], is about 915,000 in the camps. Thus, the total number of infants born in a year would be about 33,000.

### 2.2. Sample Size and Sampling

We calculated the sample size for the prevalence survey with finite population correction. As the prevalence of eye problems among the population of interest was unknown, considering prevalence as 50%, the precision of 5%, population size as 33,000 (estimated based on crude birth rate [17]) and confidence level at 95%, the estimated sample size obtained was 380. Estimating the design effect as 1.75, we got the final sample size as 665.

For sampling, we used cluster sampling method. There are two refugee settlements located at two different sub-districts (locally called ‘Upazila’) of Cox’s Bazar, a coastal district of Bangladesh. The settlement located at Ukhiya Upazila, named Kutupalong camp, is known as the largest refugee camp in the world, housing more than 630,000 Rohingya refugees. Therefore, we selected this settlement for the study.

The Kutupalong settlement is a cluster of 20 camps, and we decided to select eight camps randomly based on the coverage this project could allow and the fact that the movement of the refugees of a camp is restricted within their camp boundary. Each camp is divided into individual blocks, and eight camps consisted of a total of 44 blocks. We formed 22 clusters each consisting of two blocks. From each cluster, a frontline health worker (FHW), who is a volunteer from the refugee community, was recruited to be trained and collect data. The details of the randomisation process were as follows:

(1) To select the camps randomly, we put the 20 pieces of folded paper, each with different camp numbers (from 1 to 20) written on them, and
(2) requested one of the researchers in our team who was not involved in the study to choose eight folded pieces of paper after shaking the box each time. Hence, we selected eight camps. The characteristics of the camps are shown in Table 1:

The samples were selected by trained FHWs who took their adjacent household as the starting point and then surveyed every household clockwise in the camps to find infants matching the criteria. The inclusion criteria of our sample were as follows:

(a) The infant is 0 to 59 days old and home-based in the study area.
(b) Mother present as the primary caregiver and at least 18 years old.
(c) Mother agreed to give written consent and share information for the study.

The exclusion criteria for sampling were—

(a) Mother of an infant who does not want to give consent for the study,
(b) An infant with fever or other physical illness that may affect the examination by the FHW.

In the selected eight clusters, FHWs identified a total of 814 infants. Among them, the 22 trained FHWs interviewed 670 (82.3% response) mothers of the babies who gave consent to enrol their babies in the study and examined the babies. We surveyed from March 2019 to June 2019.

### 2.3. Survey Tools

The survey tools included the following forms: a screening form, a consent form and a questionnaire on eye problems. We prepared all forms in the Burmese language which the refugees use to read and write. The questionnaire on eye problems included closed questions along with checkboxes for answers (Yes/No) and short instructions along with each question to check the eyes of the infants according to the guideline mentioned above (a sample questionnaire is given as supplementary material). The survey form included short instructions along with each question in order to ensure identical methods of assessment during the survey.

### 2.4. Training

All FHWs were trained using the training guideline and flashcards developed for PHC providers by NCDC in 2016 [16]. The manual and the materials are endorsed by the national eye care program and used for training workers to identify and refer to eye problems of the infants nationwide.

### 2.5. Ethical Approval

We obtained ethical approval of the study from Bangladesh Medical Research Council (BMRC) – Registration number 141 14 08 2018.

### 2.6. Data Collection, Processing, and Analysis

The FHWs collected data daily. Before data collection, they were instructed to ensure if the eyes were adequately cleaned and visible. The FHWs checked and reported the following symptoms or signs for the eyes of the young infants:

(1) If the eyeball looks whitish or brownish,
(2) If there were watering or tearing from the eye while the baby is not crying and if there were any accumulation of discharge,
(3) If there is any redness present on the sclera of the eyeball.
(4) If there is any visible sign of injury present in the eye,
(5) If there was any structural deformity of the eye present in the infant,
(6) If the mother reports any problem of normal vision for her child (whitish pupillary reflex on examination),
(7) If any visual inattention is present by asking mothers if the child looks at her face and smiles.
(8) For any symptom or sign found present, the workers verbally referred the mother to the nearby health facility.

The data forms were weekly collected from the FHWs by a field coordinator, quality checked and sent to the main office to be checked, entered, cleaned and validated. After all data entry and validation processes, we analysed the data using IBM SPSS Statistics 21. We conducted the univariate and bivariate analysis for this study. To find the association between variables, we used the chi-square test with risk ratio estimate.

## 3. Results

The age of the infants ranged from 1 day to 49 days (M = 36.4, SD = 7.5) with 51.2% identified as boys and 48.8% as girls. The distribution of the surveyed infants over the camps are shown in Table 2:

The most common problem among the infants was watering from the eye (14.8%, 95% CI: 12.2–17.7). Visual inattention was reportedly found as the second most common problem in the infants reported by mothers (5.1%, 95% CI: 3.5–7.0). Also, the redness of the eye was prevalent in 4% of infants (95% CI: 2.7–5.8). An almost similar percentage of children were found to have whitish or brown eyeballs (1.9%, 95% CI: 1.0–3.3) and problem in normal vision (1.8%, 95% CI: 0.9–3.1). The health workers observed directly whitish or brown eyeballs in the infants, while mothers reported that they thought their children had a problem in normal vision. Very few children had been found with structural deformity (0.6%, 95% CI: 0.2–1.5). None of the children had any sign of injury in their eyes (95% CI: 0–0.5). Table 3 below shows details of the prevalence against each checked symptoms or signs:

We found no significant difference in the prevalence of any of the eye problems between boys and girls. Mother’s education level or age had no association with any of the eye problems among the infants.

## 4. Discussion

The most common problem found in the infants in this study was watering from the eyes which have been found common in other studies as well [19,20,21] and can be caused by a variety of problems [22]. Also, in a population study where 20% children were found to have this abnormality, in almost all cases (95%) the onset of watering from the eye was during the first month of age [21]. Nasolacrimal duct (NLD) obstruction is considered to be the most common diagnosis for watering from eyes with discharge among infants [23]. Also, the canalisation of the NLD is a common occurrence during the first month of life [24]. According to a recent book published, 30% of the infants may have watering of the eye which can be easily cured. The study also showed that 96% of the cases are resolved spontaneously [21]. However, if the condition is left untreated, it may lead to prolonged nasolacrimal duct impotency and the complications of secondary infection [19]. Therefore, a structured referral mechanism may need to be established in refugee setting for better examination of infants’ eyes.

A variety of causes can cause visual inattention in infants [25], and ophthalmologists can confirm the diagnosis. In our study at the field level, the eye problems identified validates the need for further investigation of the infants by clinically trained care providers or ophthalmologists in the refugee camps. As blindness among children and adults is a common problem in most, if not all, refugee communities, inadequate eye care services and a scarcity of literature on eye problems lead to generating less stimulation and involvement of the donors and funders to take initiatives to prevent blindness of growing children [26]. Our project trained and enabled the community members to work within their communities and identify signs for eye problems in the refugee children. Similarly, to identify cases of blindness in children of the host country key informants method had been used, validated and succeeded in identifying blindness in children [5,8,27]. The present study suggests that this approach can be applied in the refugee context as well to identify and prevent cases of childhood blindness.

Studies in other refugee camps in a resource-constrained country such as Uganda [13], Pakistan [12] addressed the prevalence of eye impairments, eye diseases, and blindness in the refugees. The prevalence survey in Pakistan among Afghan refugees found that the leading cause of blindness was cataract and uncorrected refractive errors [12] which could easily be prevented if detected earlier at a younger age. To address the need for a vulnerable population, comprehensive vision screening, improved access to eye care centres and creating evidence-based guidelines are essential [28]. Our findings from this study reinforce the idea in the Rohingya refugee camps in Bangladesh as well.

## 5. Conclusions

The prevalent eye problems demand eye care set up for the screening of eye problems in the camps with proper referral and availability of referral centres with higher service in the subdistricts and districts. This study validates the need to revisit screening facilities for common eye problems inside camps and provide the community with options to avail eye care referral services provided at higher facilities within the districts.

## 6. Limitation

The study could not follow up the referred cases and ensure the required eye care of the screening positive infants with eye problems. The reason for the higher prevalence of watering from the eye was not investigated whether it is ophthalmia neonatorum or not and any preventive measures could not be formulated.

## Figures and Tables

**Table 1:** Characteristics of camps surveyed.

Selected Camp No.	Total Population*	Total No. of Women of Reproductive Age *	No. of FHWs Assigned
2	29,918	7221	2
4	32,115	7622	4
5	25,117	5939	2
10	32,963	7791	4
11	31,346	7249	2
13	41,735	9819	2
14	31,917	7301	2
15	49,443	11,542	4

* UNHCR Population Data - 31/03/19 [18].

**Table 2:** Distribution of infants (0–59 days) among clusters.

Camp No.	Number of Infants (0–59 Days)	Percentage (%)	Boys (n)	Girls (n)
3	63	9.4	31	32
4	123	18.4	56	67
5	60	9.0	37	23
10	93	13.9	42	51
11	86	12.8	41	45
13	63	9.4	33	30
14	55	8.2	26	29
15	127	19.0	77	50
**Total**	**670**	**100**	**343**	**327**

**Table 3:** Prevalence of eye conditions among refugee infants (0–59 days) and proportions among boys and girls.

Indicators for Eye Problems	N	Prevalence (95% CI)	Boys, n (%)	Girls, n (%)
Watering from eye or accumulation of discharge	99	14.8 (12.2–17.7)	56 (16.3)	43 (13.1)
Visual inattention	34	5.1 (3.5–7.0)	21 (6.1)	13 (4)
Redness of eye present	27	4 (2.7–5.8)	15 (4.4)	12 (3.7)
Eyeball whitish or brown	13	1.9 (1.0–3.3)	9 (2.6)	4 (1.2)
Problem with normal vision (whitish pupillary reflex)	12	1.8 (0.9–3.1)	8 (2.3)	4 (1.2)
Structural deformity	4	0.6 (0.2–1.5)	1 (0.3)	3 (0.9)

## Supplementary Materials

The following are available online at https://www.mdpi.com/2414-6366/5/1/21/s1, Table S1: Eye Questionnaire for 0-59 days of Rohingya infants.
