# Supplementary material for: Prevalence of Eye Problems among Young Infants of Rohingya Refugee Camps: Findings from a Cross-Sectional Survey
Source: Trop Med Infect Dis. 2020 Feb 4;5(1):21. doi: 10.3390/tropicalmed5010021 (PMC7157546; doi:10.3390/tropicalmed5010021)
Supplement: Supplementary file 1 [file tropicalmed-05-00021-s001.pdf]

ark foundation
advancement through research and knowledge

သုညအပတ်မှ ခြောက်အပတ် အရွယ်ကလေးများအတွက် ကြည့်ရှု မေးမြန်းရမည့် မေးခွန်းလွှာ

[redacted]

စဉ် SL	လက္ခဏာများ Sign/ Symptoms	မေးမြန်းစစ်ဆေးခြင်း Ask/ Examine	အဖြေများ Results
၁<br>1	ကလေးအား ၃၅ ပတ် မတိုင်မီ မွေးဖွားခဲ့သလား<br>Was the child born before 35 weeks?	၀-၂ လ ကလေးအတွက် မိခင်အား ကလေး ၃၅ ပတ် မတိုင်မီမွေးဖွား ခဲ့သလားဟု မေးမြန်းပါ<br>For 0-2 months child, Ask the mother whether the child was born before 35 weeks?	☐ ဟုတ Yes<br>☑ မဟုတ No
၂<br>2	မျက်လုံးသည် အဖြူရောင်(သို့မဟုတ်) အညို ရောင်နှင့် တူပါသလား<br>Does the eye ball seem whitish or brown?	မျက်လုံးတစ်လုံးလုံး အဖြူရောင်(သို့မဟုတ်) အညိုရောင်ကဲ့သို့ ရှိ/မရှိ လက်နှိပ်ဓာတ်မီးဖြင့် စစ်ဆေးကြည့်ပါ။<br>Examine with torch whether any eye ball is whitish or brown	☐ ဟုတ Yes<br>☑ မဟုတ No
၃<br>3	မျက်စိမှမျက်ရည်(သို့)မျက်ချေးဆွက် ပါသလား<br>Watering from eye or accumulation of discharge.	မျက်စိနှစ်လုံးစလုံး၏ ထောင့်မှ မျက်ရည်(သို့) မျက်ချေးထွက်မှု ရှိ/မရှိ စစ်ဆေးကြည့်ပါ။<br>Examine for watery discharge or accumulation of discharge at corner of both eyes	☑ ဟုတ Yes<br>☐ မဟုတ No
၄<br>4	မျက်စိနီရဲ ပါသလား<br>Is redness of eye present?	မျက်စိနီရဲမှု ရှိ/မရှိ စစ်ဆေးပါ။<br>Examine for presence of redness of eye	☐ ဟုတ Yes<br>☑ မဟုတ No
၅<br>5	မျက်လုံးထဲတွင် ဒဏ်ရာလက္ခဏာတစ်ခုခု ရှိနေပါသလား<br>Is there any sign of injury present in eyeball?	မျက်လုံးထဲတွင် ဒဏ်ရာလက္ခဏာ ရှိ/မရှိ လက်နှိပ် ဓာတ်မီးနှင့် စစ်ဆေးကြည့်ပါ။<br>Examine with torch for presence of any sign of eye injury.	☐ ဟုတ Yes<br>☑ မဟုတ No

၆<br>6	မျက်စိစောင်းခြင်း ရှိပါသလား<br>Is squint present?	မျက်စိစောင်းခြင်း ရှိ/မရှိ စစ်ဆေးကြည့်ပါ<br>Examine for presence of squint	☐ ဟုတ Yes<br>☑ မဟုတ No
၇<br>7	ကိုယ်လက်အင်္ဂါ ချွတ်ယွင်းချက် တစ်ခုခု ရှိပါသလား<br>Is there any structural deformity?	ကိုယ်လက်အင်္ဂါ ချွတ်ယွင်းချက်ရှိ/မရှိ စစ်ဆေးကြည့်ပါ။<br>Examine for presence of any structural deformity.	☐ ဟုတ Yes<br>☑ မဟုတ No
၈<br>8	သာမန်အမြင်အာရုံတွင် ပြဿနာတစ်ခုခု ရှိပါသလား<br>Is there any problem in normal vision?	သာမန်အမြင့်အာရုံတွင် တစ်စုံတစ်ခုသော ပြဿနာ ရှိ/မရှိ ကို မိခင်ထံမှ မေးမြန်းပါ။<br>Ask the mother for any problem in normal vision.	☐ ဟုတ Yes<br>☑ မဟုတ No
၉<br>9	မျက်စိနှင့်ဆိုင်သော အာရုံစူးစိုက်မှု မရှိခြင်း ရပါသလား။<br>Is visual inattention present?	ကလေးသည် မိခင်၏ မျက်နှာဘက်ကြည့်ပြီး ပြုံးရွှင်မှု ရှိ/မရှိကို မိခင်ထံမှ မေးမြန်းပါ။<br>Ask the mother whether the child looks at her face and smiles?	☐ ဟုတ Yes<br>☑ မဟုတ No
